# First Report on Presence of Mitochondrial Introns in Freshwater Sponges, and Pseudogenic Evidence of Their Loss

**DOI:** 10.1007/s00239-025-10289-x

**Published:** 2025-12-03

**Authors:** Zhen Zhao, Junye Ma, Qun Yang, Gert Wörheide, Dirk Erpenbeck

**Affiliations:** 1 State Key Laboratory of Palaeobiology and Stratigraphy, Nanjing Institute of Geology and Palaeontology, Chinese Academy of Sciences, Nanjing, China; 2 University of Chinese Academy of Sciences, Beijing, China; 3 Department of Earth and Environmental Sciences, Palaeontology and Geobiology, Ludwig-Maximilians-Universität München, Munich, Germany; 4 GeoBio-Center, Ludwig-Maximilians-Universität München, Munich, Germany; 5 SNSB-Bavarian State Collection of Palaeontology and Geology, Munich, Germany

**Keywords:** group-II-intron, Mitochondrial intron, Porifera, Freshwater sponges, Numt, *Eunapius*

## Abstract

Mitochondrial introns have a patchy distribution in sponge lineages. Here, we report on the finding of a group-II-intron in *Eunapius rarus* (Demospongiae, Spongillidae), which constitutes the first report of a mitochondrial intron in freshwater sponges. Group-II-introns are self-splicing ribozymes, and are particularly rare among sponge mitochondrial genomes. The intron contains complete open reading frames (ORFs), including typical intron-encoded proteins (IEPs). Phylogenetic analysis reveals that the intron is more closely related to those found in brown algae, and distant from other sponge group-II-introns, indicating an acquisition of this intron independent from other sponges. Remarkably, the congeneric *E. fragilis* does not possess this intron in their mitochondrial genome. However, we found pseudogenic copies of the *E. rarus* group-II-intron in the nuclear genome of *E. fragilis*, which indicates patterns of group-II-intron presence and their pseudogene transposition into the nuclear genomes in sponges for the first time. Our results show that a group-II-intron must have been present in the last common ancestor of both *Eunapius* mt genomes, and subsequently lost in *E. fragilis*, rather than independent acquisition. Consequently, our findings provide an explanation for the patchy distribution of introns in sponges as a result of frequent losses, besides multiple acquisitions.

Mitochondrial introns are self-splicing ribozymes, which are divided into group-I and group-II-introns according to their secondary structure and splicing mechanisms (Michel et al. 1982). Group-II-introns are retroelements coding an RNA that catalyzes self-splicing (ribozyme), and an intron-encoded protein (IEP) with a reverse transcriptase (RT), maturase (X), DNA binding domain (D) and an endonuclease (En) (Blocker et al. 2005; Toro et al. 2007; Zimmerly and Semper 2015). Both components enable intron splicing and retromobility (Lambowitz and Zimmerly 2011). Group-II-introns originated either from RT components that inserted into pre-existing self-splicing RNA, generating new transposable DNA (Lambowitz and Belfort 1993; Wank et al. 1999; Mohr and Lambowitz 2003); or alternatively, self-splicing RNA inserted into terminal ends of retroelements (Curcio and Belfort 1996).

Group-II-introns are known from bacteria, archaea and organelles of several lineages of eukaryotes, including sponges (Porifera). While in Porifera group-I-introns are frequently reported from the mitochondrial cytochrome oxidase subunit 1 (cox1), group-II-introns are comparatively rare, e.g., in *Axinella verrucosa* Esper 1794 (cox1, Huchon et al. 2015 as *Cymbaxinella verrucosa* ), and *Acanthella acuta* Schmidt 1862 (cox1, cox2, rnl [with incomplete ORF], Lavrov et al. 2023; Ahmed et al. 2024). However, neither group I introns nor group-II-introns have been reported from freshwater sponges (Spongillida), a lineage that split off from their marine relatives about 309 million years ago (Plese et al. 2021).

We now discovered in the mitochondrial genome of the freshwater sponge *Eunapius rarus* Zhao & Ma, 2021 (in Zhao et al. 2021) a group-II-intron in cox1, which constitutes the first record of mitochondrial introns in freshwater sponges, and is one of the few records of group-II-introns in Porifera. Other *Eunapius* species including *Eunapius fragilis* (Leidy 1851), for which chromosome-level genome sequences are available, do not possess this intron in their mitochondrial genomes. However, now we found motifs of the *E. rarus* group-II-intron in chromosome 9 of the nuclear genome of *E. fragilis* indicating patterns of group-II-intron presence and transposition into the nuclear genomes in sponges for the first time.

*E. rarus* was collected in December, 2015, at Jingshan Lake (Zhenzhuquan Park), northern suburb of Nanjing, Jiangsu Province, East China at a depth of 20 cm (Zhao et al. 2021). The specimen examined in this study is deposited under voucher number NIGP174191 in the Type Collections of Nanjing Institute of Geology and Palaeontology (NIGP), Chinese Academy of Sciences. This specimen is the holotype and morphologically identified by Zhao and Ma (Zhao et al. 2021). Total DNA was extracted using the CTAB method (Saghai-Maroof et al. 1984), and the extracted DNA was used for library preparation (NEBNext^®^Ultra™DNA Library Prep Kit for Illumina). The prepared library was first subjected to quality control, and libraries that passed quality control were then sequenced using an Illumina NovaSeq at Tsingke bio company. In parallel, additional nuclear genome data of *E. rarus* was generated by Illumina (NovaSeq) sequencing with a read length of 150 bp, and Oxford Nanopore Technologies (PromethION). The second-generation sequencing generated 5.4 Gb of raw data, while the third-generation sequencing produced 4,596,709 reads with an average read length of 3,486 bp. The nuclear genome assembly was conducted using canu v1.8 (Koren et al. 2017), minimap2 (Li 2018), and pilon (Walker et al. 2014) for a draft genome. The mitochondrial genome assembly was conducted using SPAdes 3.13.0 (Bankevich et al. 2012). The assembled contigs were subsequently aligned against a closely related reference genome using BLAST 2.2.30+ (Benson et al. 2010). MITOS2 (Bernt et al. 2013) was used for initial annotation of the mitochondrial genome. The resulting mtDNA assembly of *E. rarus* had 4206.8X coverage, and is 30,672 bp long with the typical spongillid conserved set of 14 protein-coding, 2 rRNA, and 25 tRNA genes (ENA project number: PRJEB88401).

Intron sequences within cox1 were identified using the ORF finder as implemented in Geneious prime 2019.2.3 (Drummond et al. 2009). The resulting ORF was subsequently analysed with blastp (https://blast.ncbi.nlm.nih.gov/Blast.cgi) and InterProScan (Paysan-Lafosse et al. 2023) to detect conserved group-II-intron encoded protein domains (RT, X, and En, see Fig. 1), which served as diagnostic features for classification of group-II-intron (Dai et al. 2003; Huchon et al. 2015). The intron position was ascertained with reference to the cox1 sequence of the sponge *Amphimedon queenslandica*, following the approach of Szitenberg et al. (2010). The discovered mitochondrial intron in cox1 comprises 2,374 bp, starts at position 1141 (cf. Schuster et al. 2017), has a 2,166 bp ORF (start codon ATA at position 51, stop codon TAG), and contains the three domains RT, X, and En (Fig. 1A). In parallel, the nuclear genomes of *E. fragilis* as published in NCBI Genbank (GCA_963681505, BioProject PRJEB70489) and *E. rarus* were screened for copies of the *E. rarus* mitochondrial intron or intron motifs using NCBI blast and Geneious blast.

**Fig. 1:**
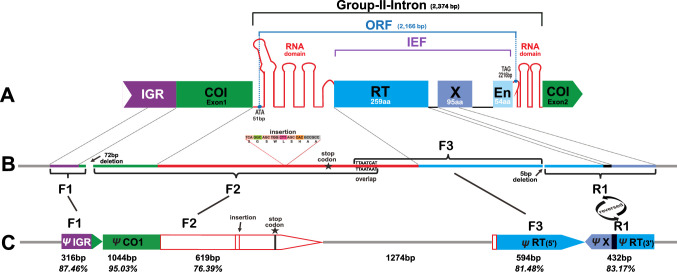
**A** Schematic view of the *Eunapius rarus* mitochondrial cox1 gene (green) with its group-II-intron (modified after Zimmerly and Semper 2015). RNA coding sections are drawn in red, IEP genes RT, X, and En in blue, purple, and light blue respectively; grey sections are noncoding. **B**, **C** Corresponding Numt fragments F1, F2, F3 and R1 in the *Eunapius fragilis* nuclear genome. **B** F1, F2, F3 and R1 mapped to the *E. rarus* mitochondrial cox1. **C** Fragment arrangement and orientation in *E. fragilis* chromosome 9. *Ψ* indicates pseudogenic copy (Numt). Lengths and nucleotide similarity to *E. rarus* mtDNA are given below the fragments

For a phylogenetic analysis of the intron sequences we downloaded intron sequences with the best hits of a Blastp match and complemented the data with group-II-intron data published from sponges totaling 55 sequences (Fig. 2). We then aligned these sequences using MAFFT v7.490 (Katoh and Standley 2013) as implemented in Geneious under default settings. The final alignment consisted of 56 taxa with 949 characters and can be obtained at LMU Open Data (10.5282/ubm/data.699). Phylogenetic analyses were performed using PhyML 3.3 (Guindon et al. 2010) for maximum likelihood (ML) with 100 rapid bootstraps under the WAG + R + F model as selected by SMS (Lefort et al. 2017). Here, the dispersed distribution of sponges and algae across different branches of the intron ORF phylogenetic tree indicates a stark difference between the ORF tree and species tree (Fig. 2). This underlines the transmission of group-II-introns both in animals, algae, and land plants not only by vertical gene transfer, but also through a combination with horizontal gene transfer (Wang and Lavrov 2008; Szitenberg et al. 2010). The *E. rarus* group-II-intron is more closely related to introns of marine brown algae, and more distant to the introns of the other—all marine—sponges, which consequently suggests that the *Eunapius* introns were acquired independently from the remaining sponges (Fig. 2). Its structural completeness could be attributed to a recent insertion, and the slow evolutionary rate of the demosponge mitochondrial genome (Shearer et al. 2002).

**Fig. 2:**
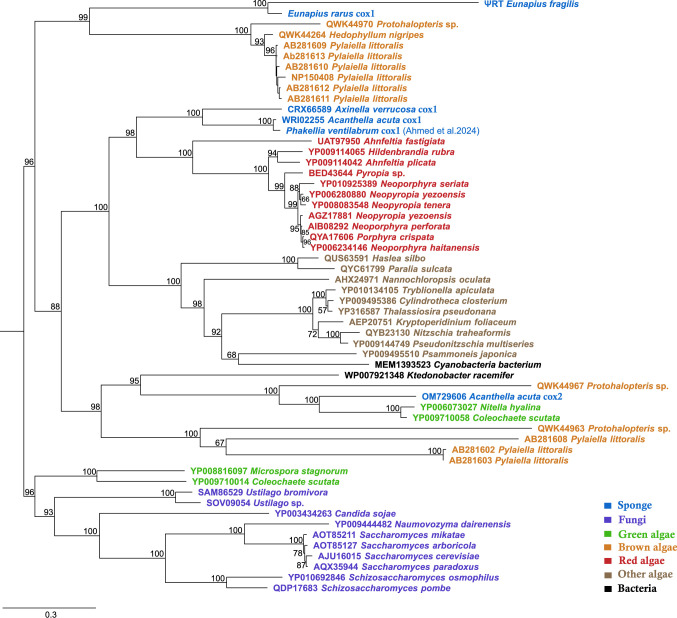
Maximum-likelihood tree of ORF from sponges and other close relative sequences published in NCBI from blast. Numbers on the branches indicate bootstrap support (> 65). Numbers preceding taxon names are Genbank accession numbers. Scale bar depicts substitutions per site

Our finding of a mitochondrial intron in a freshwater sponge is a novelty, despite their increasing number detected in other demosponge lineages (Cranston et al. 2021; Lavrov et al. 2023). Two factors foster sponge mitochondrial introns compared to other animals: First, the slow evolutionary rate of the demosponge mitochondrial genome limits the occurrence of mutations that promote intron mobility and changes in insertion sites. Except for a single intron at position 379 in the cox2 gene of *A. acuta*, all group II introns in the investigated sponges occur at position 1141 of cox1, indicating a preferential insertion into conserved regions similar to group I introns (Swithers et al. 2009; Schuster et al. 2017). Secondly, intron dispersal is promoted by asexual reproduction independent from germlines via budding, fragmentation or gemmulation, as particularly characteristic in Spongillida (Szitenberg et al. 2010; Manconi and Pronzato 2016).

Nevertheless, the phylogenetic distribution of mitochondrial introns in demosponges is patchy (Huchon et al. 2015; Cranston et al. 2021). *Eunapius fragilis* is congeneric to *E. rarus*, but its published mitochondrial genome (OY811994) lacks this group-II-intron. Whether this patchiness can be attributed to multiple, recent intron acquisitions or losses is ambiguous. However, we found two clusters of four fragments matching 87.4% the *E. rarus* group-II-intron in the *E. fragilis* nuclear genome (INSD OY811979, Fig. 1C), constituting three forward (F1-F3) and one inverted (R1) fragments, in respect to the *E. rarus* group-II-intron, separated by deletions and a 1274 bp noncoding region (Fig. 1B). They comprise the 5’ RNA-domain, RT, and partial X of the *E. rarus* intron, but also include the cox1 exon1 and 316 bp 5’ intergenic region, while En and the 3’ RNA-domain are lacking. Multiple stop codons are present, therefore, the fragments will constitute a pseudogene, rather than an active group-II-intron. The phylogenetic analysis recovers the *E. fragilis* fragments sister to the *E. rarus* group-II-intron (Fig. 2). In combination with a nucleotide similarity of 81.48–99.03%, a common ancestry rather than independent acquisition is likely.

Consequently, a group-II-intron must have been present in the last common ancestor of *E. rarus* and *E. fragilis*. In *E. fragilis*, a fragment of the mitochondrial genome has been copied and inserted in the nuclear genome as a nuclear pseudogenic fragments (Numts), as previously reported from sponge nuclear genomes (Erpenbeck et al. 2011). The mitochondrial copy of the *E. fragilis* mitochondrial intron became subsequently lost (Goddard and Burt 1999; Emblem et al. 2014; Zimmerly and Semper 2015), and contributed to the current patchy distribution of mitochondrial introns in sponges.
